# Flexible broncoscopy in patients in supportive therapy with oxygenation by extracorporeal membrane

**DOI:** 10.31744/einstein_journal/2022AO6666

**Published:** 2022-05-16

**Authors:** Camila França Redivo, Evelise Lima, Anarégia de Pontes Ferreira, Paulo Rogério Scordamaglio, Silvia Vidal Campos, Yeh-Li Ho, Ascédio José Rodrigues

**Affiliations:** 1 Universidade de São Paulo Faculdade de Medicina Hospital das Clínicas São Paulo SP Brazil Instituto do Coração (InCor), Hospital das Clínicas, Faculdade de Medicina, Universidade de São Paulo, São Paulo, SP, Brazil.; 2 Universidade de São Paulo Faculdade de Medicina Hospital das Clínicas São Paulo SP Brazil Hospital das Clínicas, Faculdade de Medicina, Universidade de São Paulo, São Paulo, SP, Brazil.

**Keywords:** Extracorporeal membrane oxygenation, Bronchoscopy, Respiratory distress syndrome, Intensive care units

## Abstract

**Objective:**

To report the experience of performing bronchoscopy in patients who underwent supportive therapy with extracorporeal membrane oxygenation in whom the bronchoscopy was performed.

**Methods:**

This was a review of medical records of patients diagnosed with extracorporeal membrane oxygenation and who required diagnostic or therapeutic bronchoscopy. Records included were related to patients admitted to the intensive care unit of *Hospital das Clínicas* of *Faculdade de Medicina* of *Universidade de São Paulo*, between 2014 and 2020.

**Results:**

During the study, 16 bronchoscopies were performed in 8 patients admitted to the intensive care unit and who underwent supportive therapy with extracorporeal membrane oxygenation. The mean age of patients was 28.37 years. Four patients were women (50%). A total of 5 (31.25%) therapeutic bronchoscopies and 11 (68.75%) diagnostics were performed. In 5 of patients, material was collected: 4 samples of bronchoalveolar lavage, three collections of transbronchial biopsies, and 1 of endobronchial biopsies. No patient had radiological worsening or hemodynamic complications. One patient (6.25%) had transient desaturation. There was moderate bleeding after transbronchial biopsy in 1 (6.25%) procedure, which was resolved endoscopically.

**Conclusion:**

Patients undergoing extracorporeal membrane oxygenation can safely perform diagnostic or therapeutic bronchoscopy provided that they have a detailed indication. Procedures were performed by a specialized bronchoscopy team in intensive care environment and with the assistance of a qualified multidisciplinary team in membrane oxygenation therapy extracorporeal.

## INTRODUCTION

Extracorporeal membrane oxygenation (ECMO) is an extracorporeal life support modality used in life-threatening respiratory and cardiac failure. The main indications for ECMO in respiratory failure are adult respiratory distress syndrome (ARDS), pneumonia, trauma, and primary graft rejection after lung transplantation. In heart failure they might be severe heart failure, cardiotomy with inability to wean off ECMO, and primary graft rejection after heart transplant. The main purpose of ECMO is to perform gas exchange (supply oxygen and remove carbon dioxide) and ensure circulatory support, besides allow protective/ultra-protective ventilation.^(1-3)^

The ECMO circuit is composed of vascular access, blood pump and gas exchange device. A dialysis membrane may be added for cases requiring renal replacement therapy. The vascular access may be venovenous (VV), when blood is drained and returned to the venous system, providing only respiratory support. Extracorporeal membrane oxygenation is indicated in cases of respiratory failure with preserved cardiac function, or venoarterial (VA), and when blood is drained from the venous system and returned to the arterial system. The use of ECMO can also provide respiratory and cardiac support in cases of combined cardiopulmonary failure.^(2,3)^

Thrombus formation in the circuit with risk of embolization is one of the main complications of ECMO. Therefore, systemic anticoagulation with unfractionated heparin is recommended. However, its use increases bleeding events, makes the balance between hemostasis and thrombosis one of the main challenges in the management of patients on ECMO. Other known complications are related to cannula insertion sites and in the ECMO system (oxygenation membrane failure and circuit breakage).^(4)^

Patients in supportive therapy with ECMO may present pulmonary complications (pneumonia, hypersecretion, pulmonary atelectasis, pneumothorax with alveolus pleural fistula) that require diagnostic and/or therapeutic bronchoscopy.^(5)^ Considering the severity of patients, invasive mechanical ventilation (IMV) and full anticoagulation, the performance of bronchoscopy may cause severe disturbances such as hemodynamic alterations, worsening of hypoxemia and pulmonary bleeding. Bronchoscopy has relative contraindication among these patients, especially when associated with bronchoalveolar lavage (BAL) and biopsies. For this reason, in such unique situations, after considering the risk/benefit of the exam, bronchoscopy should be performed in an intensive care unit (ICU) environment by a specialized team that is prepared for possible problems.^(6)^

## OBJECTIVE

To report the experience of performing bronchoscopy in patients on supportive therapy with extracorporeal membrane oxygenation.

## METHODS

This was a retrospective observational study conducted with patients admitted to the ICU of the *Hospital das Clinicas* da *Faculdade de Medicina da Universidade de* *São Paulo* (HC/FMUSP), São Paulo, between 2014 and 2020. We included patients who underwent supportive treatment with ECMO and required emergency bronchoscopy. The exams were performed by an attending physician from the Respiratory Endoscopy Service of the *Instituto do Coração do Hospital das Clínicas da Faculda de Medicina, Universidade de São Paulo* (InCor/FMUSP). We used for the exam an Olympus^®^BF-PE2 fibrobronchoscope, Plano, Texas, the United States, with a 4.9mm working channel. The technique and procedures performed during each exam were chosen according to the criteria of the attending physician.

We included patients from the ICU on supportive care with ECMO and who underwent bronchoscopy.

From the analysis of the medical records of included patients, demographic data and information related to the type of exam (diagnostic or therapeutic), the procedures performed and the complications (desaturation, hemodynamic instability, radiological alteration and airway bleeding) were collected. Desaturation was considered as a drop in peripheral oxygen saturation to values below 90%. Airway bleeding was classified as mild (aspiration only required), moderate (adrenaline use) or severe (bronchial blocker use).^(7)^

A descriptive analysis was made on considering the demographic data of the population included in the study, the type of examination, the procedures performed, and the complications observed as a result of bronchoscopy.

The present study was approved by the Ethics in Research Committee of the Institution *Instituto do Coração* (Incor) of the *Hospital das Clínicas* of the *Faculdade de Medicin*a of the *Universidade de São Paulo*, # 4,285,908, CAAE: 36968720.2.0000.0068.

## RESULTS

During the period of study, 16 bronchoscopies were performed in 8 patients admitted to the ICU and who underwent supportive therapy with ECMO. All patients were on continuous anticoagulation with unfractionated heparin. The mean age of the patients was 28.37 years. Four patients were women (50%). A total of 5 (31.25%) therapeutic bronchoscopies were performed: 3 for occlusion of pulmonary segment for treatment of high output alveolopleural fistula, and 1 for removal of obstructive clot in the bronchial tree (Table 1).

**Table 1:** Bronchoscopy indications

Bronchoscopy		n (%)
Total bronchoscopy		16 (100)
Therapeutic bronchoscopy		5 (31.25)
	Endobronchial valve insertion	1
	Application of absorbable hemostat (Gelfoam^®^)	1
	Passage of endobronchial blocker	1
	Insertion of double-lumen orotracheal tube	1
	Removal of clot from bronchial tree	1
Diagnostic bronchoscopy		11 (68.75)
	BAL collection	1
	BAL and TB collection	3
	EB collection	1
	Diagnosis of pulmonary atelectasis	1
	Diagnosis of bronchopleural fistula	2
	Check double lumen orotracheal tube	1
	Check endobronchial valve location	1
	Airway inspection	1

BAL: bronchoalveolar lavage; TB: transbronchial biopsy; EB: endobronchial biopsy.

Eleven (68.75%) diagnostic bronchoscopies were performed. Of these, 2 were conducted to elucidate pulmonary atelectasis, 2 to diagnose alveolopleural fistula, 1 to pass a double-lumen orotracheal tube, 1 to check the location of an endobronchial valve, and 1 for airway inspection. In 5 of them, material was collected: 3 collections of BAL and transbronchial biopsies, 1 collection of BAL, and 1 collection of endobronchial biopsies.

There were no hemodynamic complications or radiological worsening in any patient. There was transient desaturation in 1 (6.25%) patient. Moderate bleeding after transbronchial biopsy occurred in 1 (6.25%) procedure, which was resolved endoscopically with instillation of ice-cold saline and topical adrenaline solution (1:20,000). Five patients died during hospitalization. No deaths were related to the bronchoscopy. A description of the cases is shown in table 2.

**Table 2:** Description of reported cases

Case	Sex	Age (years)	Bronchoscopy (n)	Broncoscopy indicaiton	Days after ECMO	Complications
1	Male	53	1	Diagnosis	*	None
2	Male	35	1	Diagnosis	3 days	None
3	Female	13	4	Diagnosis: 3 Therapeutics: 1	2 days	None
4	Male	32	4	Diagnosis: 1 Therapeutics: 3	4 days	None
5	Female	16	3	Diagnosis	6 days	None
6	Female	24	1	Diagnosis	16 days	Moderate bleeding
7	Female	13	1	Therape utics	2 days	Transitory desaturation
8	Male	41	1	Diagnosis	8 days	None

* Unknown.

ECMO: extracorporeal membrane oxygenation.

### Case 1

This was a 53-year-old man, hypertensive, diabetic and obese. He was admitted to the ICU due to septic shock of pulmonary focus and severe ARDS, being submitted to supportive therapy with VV ECMO. For diagnostic elucidation, bronchoscopy with BAL collection and transbronchial biopsies were requested. There were no complications during the procedure. There was growth of *Pneumocystis jirovecii* in the BAL, and histology showed organizing pneumonia. The patient progressed with multiple organ dysfunction and died.

### Case 2

This was a 35-year-old man who was recently diagnosed with human immunodeficiency syndrome. He was admitted for progressive dyspnea, dry cough and weight loss. He presented bilateral consolidations on chest computed tomography (CT). Outpatient treatment with antibiotic therapy for community-acquired pneumonia with no improvement. He progressed to severe ARDS requiring supportive therapy with VV ECMO. For diagnostic elucidation, bronchoscopy with BAL and transbronchial biopsies were performed. There were no complications during the procedure. The polymerase chain reaction (PCR) was positive for *P. jirovecii* in the BAL and the pathological examination was positive for *P. jirovecii*. The patient received directed treatment, with improvement of the clinical picture.

### Case 3

This was 13 years old girl without comorbidities. She was admitted to the ICU due to ARDS caused by influenza B. She evolved with pneumonia associated with IMV, septic shock and pleural empyema due to Stenotrophomonas maltophilia, requiring supportive therapy with VV ECMO. Bronchoscopy was requested because of suspicion of a high output bronchopleural fistula, which confirmed the diagnosis of a fistula in the middle lobe. This was subsequently treated endoscopically with insertion of a size 5.5 unidirectional endobronchial valve (Zephyr^®^) in the middle lobe to occlude the fistula. During the clinical evolution, 2 more bronchoscopies were necessary, 1 to check the location of the endobronchial valve and the other to inspect the airway due to extubation failure. The patient evolved with respiratory improvement and reduced air leakage. She was decannulated from ECMO in 2 days and disconnected from IMV after 15 days. There were no complications related to bronchoscopy. The endobronchial valve was removed on an outpatient basis 3 months later.

### Case 4

This was a 32 years old man with a recent diagnosis of acquired human immunodeficiency syndrome. He was admitted to the ICU for pulmonary infection by *P. jirovecii*, spontaneous pneumothorax and refractory hypoxemia requiring supportive therapy with VV ECMO. Bronchoscopy was requested for suspected high output bronchopleural fistula, which confirmed the diagnosis of lingula fistula. Therapeutic bronchoscopy was performed with occlusion of the lingula with absorbable hemostatic agent (Gelfoam^®^) with transient improvement. Another bronchoscopy was performed with occlusion of the lingula using an Arndt^®^ 7F endobronchial balloon, also with transient success. Finally, the simple orotracheal tube was replaced by a double lumen tube and independent pulmonary ventilation guided by bronchoscopy. There were no complications in any of the bronchoscopies performed. The case of the patient remained without resolution of the bronchopleural fistula, and he also presented multiple infections and hemodynamic worsening, and died.

### Case 5

This was a 16-year-old female girl with cystic fibrosis presented in the fifth month of bilateral lung transplantation. She was hospitalized for pulmonary infection by Rhizopus sp. and evolved with severe ARDS and need for supportive therapy with VV ECMO. Patient progressed with pulmonary atelectasis, and 2 diagnostic bronchoscopies were performed which showed no airway changes. Because the clinical picture worsened, diagnostic bronchoscopy was requested with collection of BAL and transbronchial biopsies to support in antimicrobial treatment. During bronchoscopy, whitish plaques were visualized in tracheobronchial mucosa. Transbronchial biopsy showed fungal infection and there was growth of *Cunninghamella ssp* in BAL, leading to change of management and specific treatment for the fungus found. There were no complications in any of the bronchoscopies performed. The patient died 1 month later after lung retransplantation.

### Case 6

This was a 24-year-old obese pregnant woman at 27 weeks gestational age. She was admitted to the ICU for severe ARDS caused by severe acute respiratory syndrome coronavirus 2 (SARS-CoV-2). The patient evolved with fetal distress and underwent cesarean section. She maintained refractory hypoxemia after protective IMV, and supportive therapy with VV ECMO was indicated. Diagnostic bronchoscopy with bronchial lavage and transbronchial biopsy was requested for evaluation of possible superimposed pulmonary infection. Bronchial lavage was also performed, and thick secretion cork was removed. After the transbronchial biopsy, there was moderate bleeding, which resolved with instillation of ice-cold saline and adrenaline solution (1:20,000). There were no other complications. There was no microbial growth on the BAL, and histology resulted in organizing pneumonia. The patient developed refractory septic shock 1 week later and died.

### Case 7

This was a 13-year-old girl, postoperative of emergency cesarean section due to premature labor (27 weeks). The patient evolved with hypovolemic shock, suspected chorioamnionitis and acute respiratory failure secondary to pulmonary edema due to hypervolemia and accidental pneumothorax to the left. After orotracheal intubation and chest drainage, there was no pulmonary re-expansion. The patient evolved with hypoxemia and peripheral oxygen saturation of 48%, and underwent supportive therapy with VV ECMO. Bronchoscopy was requested, and revealed a large organized clot partially obstructing the left main bronchus and completely obstructing the left upper lobe bronchus. After bronchial clearance with fragmentation of the clot on prolonged examination (about 90 minutes) with some episodes of oxygen desaturation, there was pulmonary reexpansion. The patient was decannulated from ECMO the next day and extubated 3 days after bronchoscopy.

### Case 8

This was a 41-year-old previously healthy man and who was admitted to the ICU for severe ARDS caused by SARS-CoV-2. Because of refractory hypoxemia, supportive therapy with VV ECMO was required. He evolved with spontaneous pneumothorax in the right hemithorax, hemothorax after a pleural drain, and a high output bronchopleural fistula. During diagnostic bronchoscopy, a large amount of purulent secretion and extrinsic compression of the right lower lobe were observed. Bronchoalveolar lavage was performed in the middle lobe, without complications during the procedure. The BAL showed growth of *Klebsiella aerogenes* and Pseudomonas putida. The patient died of refractory septic shock and multiple organ and system failure.

## DISCUSSION

The use of ECMO as a supportive therapy in patients with cardiorespiratory failure is on the rise. Improvements in ECMO technology and management of critically ill patients, including protective mechanical ventilation in ARDS, have allowed increased survival in this population.^(8,9)^ The increased survival, complications, especially those related to nosocomial infections, have been observed.^(9)^ The need to identify pathogens causing respiratory infection for a targeted therapy has made flexible bronchoscopy an indispensable procedure in these patients.^(8)^

Indications for flexible bronchoscopy in patients submitted to support therapy with ECMO may be of diagnostic or therapeutic nature. The main ones are related to diagnostic support in pulmonary infections, many times with need for collection of samples from the respiratory tract (BAL and transbronchial biopsy), bronchial hygiene, and evaluation of pulmonary atelectasis and airway patency.^(10)^

However, the risk of complications associated with bronchoscopy should not be underestimated, since patients are critically ill, under IMV, anticoagulated and most of the time present severe pneumopathy. Such situation would culminate in indication for ECMO given that the main indications for ECMO are related to bacterial pneumonia, viral pneumonia and ARDS secondary to postoperative or trauma.^(11)^ The most feared complication is pulmonary bleeding, since spontaneous bleeding in patients under ECMO is 8.1%, and this value could increase to 19% in patients undergoing bronchoscopy. Other possible complications are displacement of the ECMO cannula, loss of ECMO flow and accidental extubation.^(5,12)^

Retrospective studies have shown bronchoscopy to be a well-tolerated, safe procedure with a low complication rate in patients undergoing ECMO support therapy in both pediatric and adult populations.^(5,10,13,14)^

In this case series, 16 flexible bronchoscopies were performed in 8 patients. Eleven (68.75%) were performed for diagnostic purposes. Bronchoscopy for diagnostic support of lung infection was performed in 45.45% (n=5), and there was pathogen growth in 60% (n=3) of them. Five (31.25%) bronchoscopies were performed for therapeutic purposes. In 2 patients there was a need for treatment of a high output alveolopleural fistula. One patient was successfully treated with insertion of a size 5.5 unidirectional endobronchial valve (Zephyr^®^). All patients were in critical condition and underwent bronchoscopy with careful indication. No major complications were observed. Minor complications occurred in 2 (12.5%) procedures, and they were promptly treated. In 1 case, there was moderate bleeding and in another, transient desaturation. Desaturation during VV ECMO support, although not expected, may occur due to several mechanisms (recirculation rate, pulmonary shunt, cardiac output, ECMO blood flow and oxygenator dysfunction).^(15)^

Although bronchoscopy helped in the management of all cases presented, only 3 (18.75%) patients survived. This is probably due to the fact that initially the use of VV ECMO was reserved for extremely severe patients. However, a change in this perspective was observed due to the increased availability and dissemination of technology of this therapeutic modality.

This study highlights the number of bronchoscopies performed in patients undergoing VV ECMO, which is higher than in most published articles, the low complication rate, and the positive impact on case management.

This study has limitations. It was retrospective, with a small number of patients included, and with information collected from medical records, which may be understood as incomplete.

## CONCLUSION

Patients on extracorporeal membrane oxygenation can safely undergo diagnostic or therapeutic bronchoscopy; The procedure should be reffered and performed by a specialized bronchoscopy team at an intensive care unit environment. In addiion, the assistance of a skilled multidisciplinary team in extracorporeal membrane oxygenation is crucial. Further and larger prospective studies should be conducted to confirm these preliminary findings.
